# Radiosensitivity of Cancer Stem Cells Has Potential Predictive Value for Individual Responses to Radiotherapy in Locally Advanced Rectal Cancer

**DOI:** 10.3390/cancers12123672

**Published:** 2020-12-07

**Authors:** Caterina Puglisi, Raffaella Giuffrida, Giuseppina Borzì, Paolo Di Mattia, Anna Costa, Cristina Colarossi, Enrica Deiana, Maria Carolina Picardo, Lorenzo Colarossi, Marzia Mare, Lorenza Marino, Alfio Di Grazia, Stefano Forte

**Affiliations:** 1 IOM Ricerca Srl, Viagrande, I-95029 Catania, Italy; caterina.puglisi@grupposamed.com (C.P.); raffaella.giuffrida@grupposamed.com (R.G.); anna.costa333@alice.it (A.C.); 2 REM Radioterapia, Viagrande, I-95029 Catania, Italy; giuseppina.borzi@grupposamed.com (G.B.); lorenza.marino@grupposamed.com (L.M.); alfio.digrazia@grupposamed.com (A.D.G.); 3 Surgical Oncology Unit, Mediterranean Institute of Oncology, Viagrande, I-95029 Catania, Italy; paolo.dimattia@grupposamed.com (P.D.M.); enrica.deiana@grupposamed.com (E.D.); carolina.picardo@grupposamed.com (M.C.P.); 4 Pathology Unit, Mediterranean Institute of Oncology, Viagrande, I-95029 Catania, Italy; cristina.colarossi@grupposamed.com (C.C.); lorenzo.colarossi@grupposamed.com (L.C.); 5 Medical Oncology Unit, Mediterranean Institute of Oncology, Viagrande, I-95029 Catania, Italy; marzia.mare@grupposamed.com

**Keywords:** cancer stem cells, locally advanced rectal cancer (LARC), neo-adjuvant radiotherapy, in vitro radiotherapy, preclinical model of radiotherapy

## Abstract

**Simple Summary:**

Radiotherapy is often used as a neo-adjuvant treatment in locally advanced rectal cancer. While treatment generally induces an improvement in the outcome, some patients show resistance to treatment for reasons that still have to be elucidated. In this work, we report an in vitro and in vivo model based on patient-derived cancer stem cells. This model is able to predict individual responses to radiotherapy. The results indicate that cells found to be radiation-sensitive in vitro generated radiation-sensitive tumor xenografts upon subcutaneous implantation. Analogously, cancer stem cells (CSCs) that did not respond to in vitro radiation treatment generated radiation-resistant tumor xenografts. Moreover, radioresistant CSCs were generally isolated via biopsies of patients with poor responses to neo-adjuvant radiotherapy. This suggests that a cell-based in vitro test may itself be sufficient to predict outcomes in donor patients.

**Abstract:**

Neo-adjuvant radiotherapy is frequently employed in the therapeutic management of locally advanced rectal cancer (LARC). Radiotherapy can both reduce local recurrence and improve the success of surgical procedures by reducing tumor mass size. However, some patients show a poor response to treatment, which results in primary resistance or relapse after apparent curative surgery. In this work, we report in vitro and in vivo models based on patient-derived cancer stem cells (CSCs); these models are able to predict individual responses to radiotherapy in LARC. CSCs isolated from colorectal cancer biopsies were subjected to in vitro irradiation with the same clinical protocol used for LARC patients. Animal models, generated by CSC xenotransplantation, were also obtained and treated with the same radiotherapy protocol. The results indicate that CSCs isolated from rectal cancer needle biopsies possess an intrinsic grade of sensitivity to treatment, which is also maintained in the animal model. Notably, the specific CSCs’ in vitro and in vivo sensitivity values correspond to patients’ responses to radiotherapy. This evidence suggests that an in vitro radiotherapy response predictivity assay could support clinical decisions for the management of LARC patients, thus avoiding radiation toxicity to resistant patients and reducing the treatment costs.

## 1. Introduction

Colorectal cancer (CRC) is the third most common cancer (10.2% of tumor diagnoses) in terms of incidence and the second most common in terms of mortality, for both sexes combined, in western countries [1]. While specific causes of CRC insurgence have not been identified, different factors have been implicated in colorectal tumorigenesis, including genetic background, age, and different lifestyle factors, such as alcohol consumption [2], smoking [3], obesity [4], physical inactivity [5], and a dietary regimen comprising low fiber intake and high consumption of red/processed meat [6,7].

Cancers of the rectum account for 30% of all diagnosed CRCs. Although rectal cancers are histologically similar to colon cancers, considerable biological and clinical evidence indicates that rectal cancer must be considered a distinct entity [8,9]. Total mesorectal excision, preceded by neoadjuvant radiotherapy or chemo-radiotherapy [10,11], is the standard treatment for LARC. This approach reduces the risk of local recurrence, although it does not increase survival compared to surgery alone [12].

Despite improvements in treatment, the prognosis of CRC remains unfavorable. Although several important side effects can occur, negatively affecting patient quality of life [13], radiotherapy has been reported to significantly promote the downsizing and downstaging of large CRCs in neoadjuvant settings [14]. Notably, only a limited number of patients affected by CRC respond positively to a neoadjuvant treatment regimen, likely due to tumor heterogeneity.

Cancer Stem Cells (CSCs) are a small subset of cells that possess self-renewal potential and persistent tumorigenic capacity. Indeed, it has been widely demonstrated that CSCs are able to initiate and sustain tumor growth and, moreover, to recapitulate cancer cell heterogeneity. The existence of CSCs was first demonstrated in acute myeloid leukemia [15] and was successively described in other hematological and solid tumors [16,17,18,19,20,21,22,23,24,25]. CSCs normally represent 0.1–10% of all tumor cells, and their identification is based on the expression of specific surface markers [26,27]. CSCs from colon cancer have been widely studied thanks to the identification of specific markers, including Lgr5 [28,29,30], CD133 [22,31], and CD44 [32,33]. In such studies, CSCs were shown to be responsible for tumor maintenance and propagation upon xenotransplantation [22,34,35].

Conventional anti-cancer therapies target only differentiated and fast proliferating cells, while sparing CSCs that are quiescent and undifferentiated. For this reason, CSCs are often drug-resistant, leading to tumor recurrence and metastasis [36]. Consequently, CSCs represent the primary therapeutic targets to abrogate the minimal residual disease and impede the reappearance of tumor lesions.

In this study, we developed an in vitro and in vivo model of radiotherapy, based on patient-derived CSCs, for the prediction of treatment efficacy to support clinical decisions. Our data indicate a concordance between in vitro and in vivo CSC sensitivity to radiotherapy, suggesting that the CSC model may be sufficient to assess the suitability of therapeutic regimens, thus providing a feasible translational approach for the prediction of efficacy. The proposed approach may be useful for the timely identification of resistant patients who can be spared from side effects of ineffective radiotherapy, thereby supporting more appropriate clinical decisions for therapeutic options.

## 2. Results

### 2.1. Molecular Phenotype of Tumor Sphere Cultures

CD44 and CD133 molecules are two common surface markers used for the identification and isolation of colorectal CSCs [22]. Flow cytometry analysis revealed a modest expression of CD133 (around 20–25%) for all four lines analyzed. Instead, CD44 showed a much higher expression for Lines 2 and 3 (70.7% and 76%, respectively), while Lines 1 and 4 revealed the expression of CD44 comparable to that of CD133 (around 21%) (Table 1).

Increased aldehyde dehydrogenase (ALDH) activity has been described in cancer stem cells from different carcinomas [19,37,38,39,40,41]. Moreover, several ALDH isoforms have been identified as CSC markers in different tumor types, including colon cancer [42]. Medium ALDH activity was found in all four lines analyzed (Table 1).

### 2.2. Animal Model-Derived Tumors Present the Same Phenotype as Human Parental Cancer

A pre-requisite of putative CSCs is their capacity to develop tumors that have the same phenotype as human parental cancer when transplanted in recipient mice.

CSCs derived from CRC biopsies were injected subcutaneously into the flanks of thymic immunocompromised mice. This procedure resulted in 100% tumor formation efficiency within a few weeks (2–4 weeks) after injection for all four cell lines. The tumor mass explanted from the animal was processed for histologic and immunohistochemical analysis to verify that its phenotypical features reproduced those of the original human tumor.

**Figure 1:**
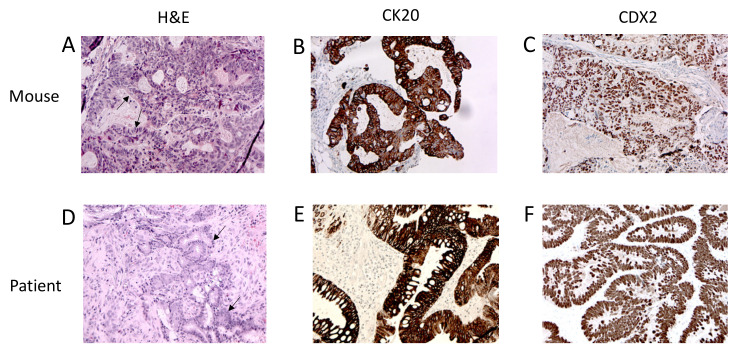
Animal model-derived tumors presented the same phenotype as human parental cancer. (**A**) Hematoxylin and Eosin (H&E) staining, (**B**) CK20 and (**C**) CDX2 immunohistochemistry of the mouse xenograft. (**D**) Hematoxylin and Eosin staining, (**E**) CK20 and (**F**) CDX2 immunohistochemistry of human colorectal cancer biopsy. Arrows indicate the gland structures. Magnification 20×.

As shown in Figure 1, the xenografts presented the histological (A) and immunohistochemical (B,C) features of human colorectal cancer (D–F), including the formation of glands (arrows) and stromal components. Similar to their human counterparts, the adenocarcinomas here showed variability in the size and configuration of their glandular structures. In well and moderately differentiated adenocarcinomas, the epithelial cells were usually large and tall and often contained cellular debris in the gland lumen. In the mouse, the xenograft glands maintained the expression of anti-human CK20 (Figure 1B) and CDX2 (Figure 1C), two typical CRC markers.

### 2.3. CSCs from Different Rectal Biopsies Showed Different In Vitro Sensitivity Levels to Radiotherapy

The establishment of CRC stem cell cultures may facilitate the direct evaluation of radiation cytotoxic activity on the putative cells responsible for tumor growth and spread, which represent optimal cellular targets for successful therapy. As a fractioned 25 Gy dose administered daily (5 Gy/Day) is the most commonly updated protocol used before proceeding with curative surgery for rectal cancer [43], the same schedule was used to treat the CSC in vitro.

**Figure 2:**
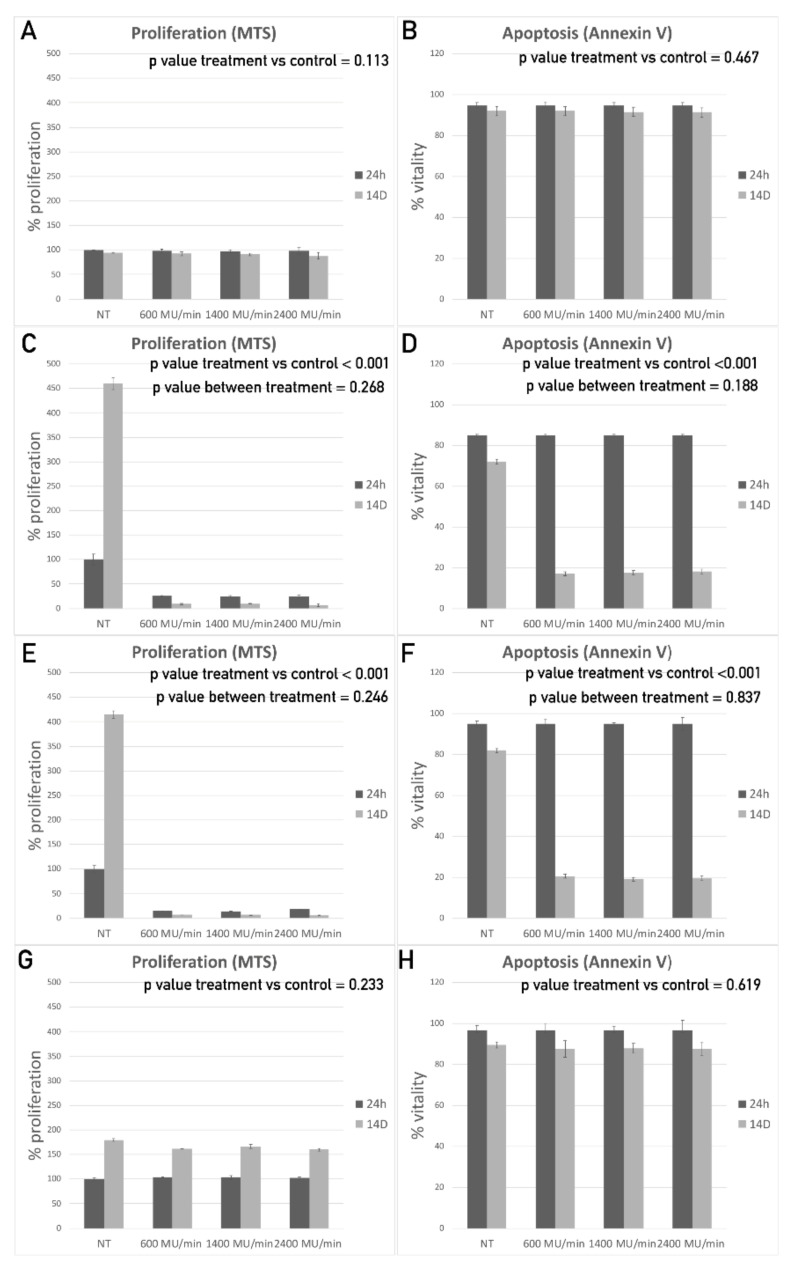
Cancer stem cells (CSCs) from different rectal biopsies show different in vitro sensitivity levels to radiotherapy. Proliferation modulation, in comparison with untreated cells, is reported for Line 1 (**A**), Line 2 (**C**), Line 3 (**E**) and Line 4 (**G**). Vitality modulation, in comparison with untreated cells, is reported for Line 1 (**B**), Line 2 (**D**), Line 3 (**F**) and Line 4 (**H**).

To evaluate the effects of the radiotherapy treatment in vitro, different assays, including proliferation, apoptosis, and single cell cloning assays, were performed on irradiated cells at different time points (24, 48, and 72 h and 7 and 14 days). Since radiation can be administered using different dose rates during the daily sessions, the effects of the dose rate in terms of cell growth arrest and apoptosis induction were investigated. In vitro cultures were treated using a 5 Gy daily dose at 600, 1400, and 2400 MU/min, and the MTS and Annexin V results were compared. No statistically significant difference was observed among the different dose rate treatments in all the lines assessed (Figure 2A–H). On the other hand, different cell lines showed significantly different overall sensitivity to the treatment. In particular, Line 1 and Line 4 CSCs displayed similar behavior, both becoming rather resistant to radiation (Figure 2A,B,G,H). Indeed, cell proliferation was not significantly reduced at any dose rate or at any time point considered for either Line 1 or Line 4, compared to the control sample (Figure 2A,G; repeated measures ANOVA (RMAN) *p* values, respectively, of 0.113 and 0.233). Accordingly, the apoptosis assay did not show any significant difference in the percentage of cell vitality between the treated and untreated (NT) samples at any dose rate or at any time point considered (Figure 2B,H; RMAN *p* value, respectively, of 0.467 and 0.619). Finally, the single cell cloning assay exhibited only a slight reduction for both lines (25% and 30%, respectively) in their ability to give rise to new cell clusters 7 days after treatment (data not shown).

On the contrary, the other two CSC lines (Lines 2 and 3) were shown to be highly sensitive to radiotherapic treatment. The proliferation assay revealed a significant reduction in cell proliferation for both lines at all dose rates tested (600, 1400, and 2400 MU/min) compared to the NT samples. In particular, after 14 days, almost all the cells were shown to have lost their proliferative potential in all treated samples (Figure 2C,E; RMAN *p* values both below 0.001 with no significant differences between dose rates). The Annexin V assay confirmed these data, already showing a reduction in cell vitality of about 80% at 14 days after treatment (Figure 2D,F; RMAN *p* values both below 0.001 with no significant differences between dose rates). Although all assays were performed on irradiated cells at five different time points (24, 48, and 72 h and 7 and 14 days), data are reported only for the most informative time points (24 h, which is the early timepoint, and 14 days, which is the latest timepoint). In accordance with the radiobiology of the treatment, the cells start to die only a few days after the treatment because they were unable to repair DNA damage and accumulated mutations. At the earlier time points, the induction of cell death was not noticeable, even when already triggered.

Seven days after radiation, both cell lines showed a 100% reduction in their clonogenic potential (data not shown).

These in vitro results demonstrated that the CSCs derived from different patients had diverse sensitivity levels to radiation treatment, although no differences were found among the different dose–rate protocols tested.

Graphical representations of the proliferation (A, C, E, G) and apoptosis assay (B, D, F, H) 24 h and 14 days (14D) after treatment with a 5 Gy dose radiation supplied for 5 consecutive days in combination with different dose rates (600 MU/min, 1400FFF MU/min and 2400 MU/min) in comparison with the non-treated sample (NT) for CSC Line 1 (A, B), Line 2 (C, D), Line 3 (E, F), and Line 4 (G, H).

### 2.4. CSCs from Different Rectal Biopsies Showed In Vivo Sensitivity to Radiotherapy Comparable to That Found under In Vitro Treatments

The individual responses of CRC patients to neoadjuvant radiation may vary in relation to the unique characteristics of each individual tumor, differently affecting the response to radiotherapy in terms of mass reduction and risk of recurrence.

In vitro experiments already demonstrated that the cells isolated from different colorectal cancer patients present different sensitivity levels to radiotherapeutic treatments. To evaluate the in vivo responses to radiotherapy, animal models were exposed to the same radiotherapy protocols used in the in vitro experiments.

**Figure 3:**
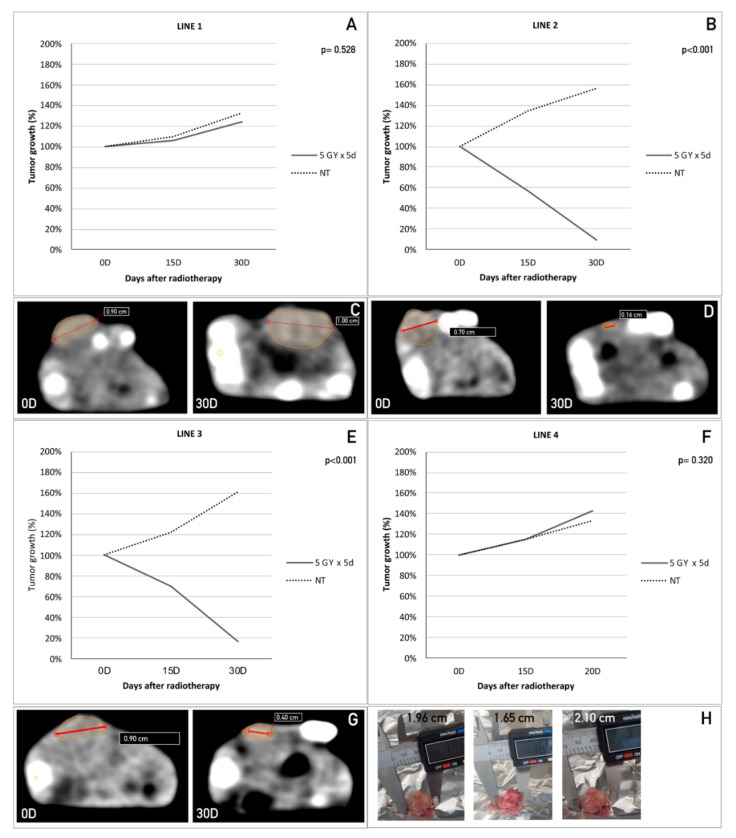
CSCs from different rectal biopsies showed in vivo sensitivity to radiotherapy comparable to that found under in vitro treatments. Graphical representations of in vivo tumor growth (%) measured at three different time points (0D, 15D, 30D) after treatment with 5 Gy dose radiation, supplied for 5 consecutive days, in combination with a dose rate of 2400 MU/min, in comparison with not treated sample (NT), for CSC Line 1 (**A**), Line 2 (**B**), Line 3 (**E**) and Line 4 (**F**). (**C**,**D**,**G**) Representative CT scan images taken the day of the last irradiation (D0) and 30 days after treatment for line 1, 2 and 3. Line 4 produce fast growing refractory tumors with consequent risk of ulceration. All mice implanted with line 4 had to be sacrificed at day 20 because they reached tumor mass limit. For this reason, CT scan images are not available. Representative images of ex vivo measures for line 4 (**H**).

To this end, cancer stem cells derived from patients with colorectal tumors were first expanded in vitro and, after reaching a sufficient amount, were injected into the flank of female athymic immunodeficient mice. Tumor growth was evaluated every 4 days using an external digital caliper. Within 2–4 weeks, all the animals showed the formation of a tumor mass for all four cell lines tested. Mice were divided into two groups: a control group (NT: not treated) composed of three animals and a treated group containing eight animals. Once the tumors reached a diameter of about 100–150 mm^3^, the animals were subjected to a TC scan to better estimate the tumor dimensions and exactly define the zone to expose to radiation. Mice were treated with 5 Gy radiation for 5 consecutive days during the same hour and under the same experimental conditions. Tumor growth was evaluated by external caliper measurements every 4 days for 4 weeks. At the end of this period, the mice were subjected to a final TC scan to exactly evaluate their tumor mass reduction in comparison with the initial TC scan. The in vivo results were fully consistent with those obtained in vitro. In particular, the radiation treatment was ineffective for Line 1- and 4-derived tumor grafts, as shown in Figure 3A,F, where the trend of tumor growth for the treated group was fully comparable to that of the NT group. The CT scan images of the tumor at the beginning (0D) and end (30D) of the treatment confirmed the data obtained with the caliper (Figure 3C) for Line 1. Line 4 produced fat-growing refractory tumors that rapidly reached the maximum volume allowed by the in vivo protocol in all the animals implanted, both treated and untreated. The animals were sacrificed 20 days after the start of the treatment. For this reason, D30 CT scans are not available for these animals. Figure 3H reports some representative ex vivo measures of the tumor masses. Conversely, Lines 2 and 3 tumor grafts displayed a high sensitivity to the treatment, revealing a steady decrease in tumor growth over time, down to zero (Figure 3B,E; RMAN *p* values below 0.001). The same trend was observed in the CT scan images at the beginning (0D) and end (30D) of the treatment (Figure 3D,G).

Ex-vivo histological analysis confirmed tumor regression in the treated explants derived from Lines 2 and 3. In these tissues, no viable tumor cells were observed. On the other hand, explanted tissues derived from treated tumors derived from Lines 1 and 4 presented viable tumor cells.

Graphical representations of the in vivo tumor growth (%) measured at three different time points (0D, 15D, and 30D) after treatment with a 5 Gy dose of radiation supplied over 5 consecutive days in combination with a dose rate of 2400 MU/min compared to the non-treated sample (NT), for CSC Line 1 (A), Line 2 (B), Line 3 (E), and Line 4 (F). (C, D, G) Representative CT scan images taken on the day of the last irradiation (D0) and 30 days after treatment for Lines 1, 2, and 3. Line 4 produced fast growing refractory tumors with a consequent risk of ulceration. All mice implanted with Line 4 had to be sacrificed on day 20 because they reached the tumor mass limit. For this reason, CT scan images are not available. Representative images of ex vivo measures for Line 4 (H).

### 2.5. In Vitro Treatment Predicted the Clinical Outcomes of Rectal Cancer Patients Treated with Neo-Adjuvant Radiation

In the clinic, all four CRC patients whose CSCs were isolated and used for the in vitro experiments were subjected to neo-adjuvant radiotherapy. In this way, we compared the in vitro responses with the clinical outcomes.

**Figure 4:**
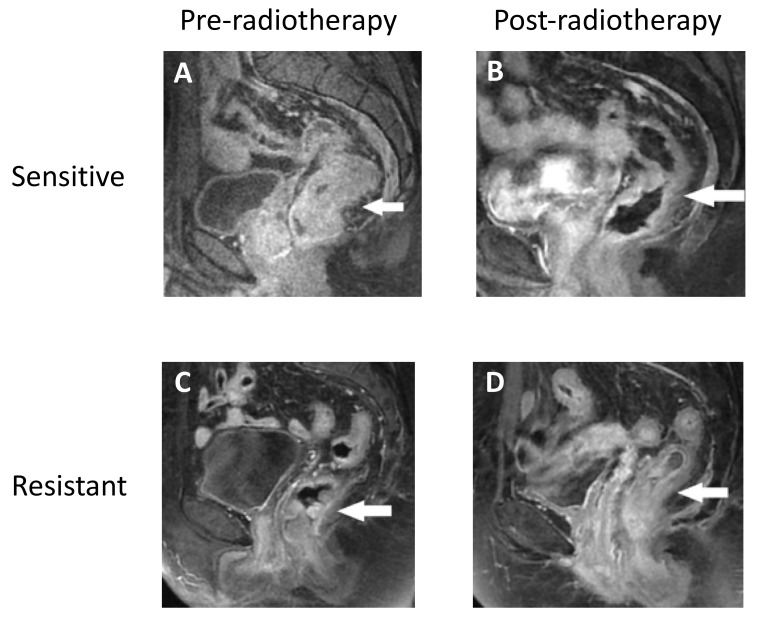
In vitro treatment predicted the clinical outcomes of rectal cancer patients treated with neo-adjuvant radiation. Representative MRI images before (**A**) and after (**B**) the radiotherapy of a patient whose CSCs became sensitive to in vitro treatment. Representative MRI images before (**C**) and after (**D**) radiotherapy of a patient whose CSCs became resistant to in vitro treatment.

Our data show that the patients achieved a good clinical response when their isolated CSCs were sensitive to in vitro treatments, as shown in Figure 4A,B, where a significant reduction in tumor mass was obtained after radiotherapy in patients whose cells became sensitive when treated in vitro. On the other hand, the patients whose CSCs became resistant to in vitro radiation presented a poor clinical response and could not be subjected to post neoadjuvant surgical treatment because of their unaltered tumor dimensions (Figure 4C,D).

## 3. Discussion

The European Society of Medical Oncology (ESMO) guidelines [44] recommend the use of neoadjuvant therapy for rectal cancer in cases classified as advanced disease (>>T3), when there is suspected lymph node involvement, or when total mesorectal excision may be impaired by the state of the circumferential resection margin. In patients with the involvement of the anal sphincter, successful tumor downsizing may allow for the creation of safe resection margins, thus facilitating the preservation of anal functionality. Moreover, it has been shown that some patients presenting a complete response to radiotherapy can be enrolled in surveillance programs without the need for surgical treatment. Various studies [45,46] have been performed to evaluate the feasibility of a watch-and-wait strategy in patients who show a complete pathological response after neo-adjuvant therapy. These studies indicate that, for a specific class of patients who show a complete response to neoadjuvant therapy, surgical treatment may be an option if a stringent surveillance strategy is used. However, a significant number of responders may be affected by local regrowth or metastatic dissemination. As previously discussed, this phenomenon is strongly associated with the capability of CSCs to resist treatment and promote cancer progression even when patients exhibit an apparent response to neo-adjuvant therapy.

As the evaluation of the complete pathological response alone is not sufficient to identify the cases that, despite an apparent initial response to the treatment, may be affected by recurrence events, thus compromising the clinical outcome, the identification of more effective strategies for the prediction of treatment efficacy is desirable. On the other hand, radiotherapy is not free from undesirable effects, as it increases the risk of serious anorectal and genitourinary complications, which may negatively impact a patient’s quality of life. Numerous radiotherapy-related side-effects have been described, including surgical site infection, anastomotic leakages, wound dehiscence, and functional disorders, such as low anterior resection syndrome and genitourinary dysfunction. The severity of these effects is affected by individual susceptibility, the dose of administered radiation, and the accuracy with which the radiation is precisely delivered to the target area. For the above-mentioned reasons, it is very important to accurately identify both the patients who are likely to benefit from neoadjuvant radiotherapy and the most suitable therapy (dose or protocol) for each individual to optimize patient management and support clinical decisions to minimize morbidity and improve clinical outcomes.

Our data suggest that heterogeneity exists in the individual sensitivity levels of patient-derived CSCs to different doses of radiation. We demonstrated that CSCs have specific individual dose thresholds, above which they are triggered to stop proliferation, activate the apoptotic pathway, and lose the stem-cell-like phenotype. The concordance between the treatment effects under both in vitro and in vivo irradiation indicates that this individual sensitivity is not affected by experimental conditions and seems to be related only to the intrinsic cellular phenotype. While the underlying cellular and molecular mechanisms responsible for these different responses are yet to be elucidated, the in vitro model itself may be proposed as an investigational tool to predict the therapeutic response of the donor patient. The experimental procedure evaluated in this work was developed to potentially fit a clinical scenario in an effort to define a protocol for personalized medicine in radiotherapy. On the one hand, we demonstrated that CSCs can be isolated from scarce materials like the biopsy tissue collected from patients before any intervention. To evaluate the feasibility of a translational protocol able to predict responses to neo-adjuvant therapy, the only material available for CSC isolation is provided by a biopsy. According to our experience, the isolation of CSCs from solid tumors [25] may be affected by both the quantity and the quality of the starting material. The protocol we developed for sampling, CSC isolation, and expansion was shown to be suitable to yield primary CSC cell cultures in most cases. Importantly, some samples did not produce vital CSC cultures. The reasons for this may be related to the individual characteristics of each tumor or to the limits of the biopsy sampling process, which may sometimes be unable to collect the appropriate cellular subpopulation.

On the other hand, to be effective, a predictive protocol must provide the clinician with relevant information quickly enough to treat the patient. While CSCs are isolated, in vitro expansion and in vitro treatment can be completed in a relatively short time, while the development of an in vivo xenotransplantation model is much more time demanding. To develop patient-derived mice models, 2.5 × 106 cells were obtained (5 × 105 cell for five mice). Then, after the subcutaneous cell implantation, the CSC-derived tumor masses required from 3 to 9 weeks to reach a sufficient volume for the subsequent experimental procedure. After the mice were treated, they were observed for about one additional month to evaluate the final effect of the treatment. In all the evaluated cases, we observed perfect concordance between the results of the matched in vivo and in vitro treatments. For each of the patient-derived CSC lines, both the CSC culture and the patient-derived mouse model were shown to possesses the same dose sensitivity threshold. While this observation is preliminary, this concordance suggests that an in vivo procedure may not be required to identify the lowest effective dose of radiation. Finally, we demonstrated that every time a patient-derived CSC culture exhibited a certain level of radio resistance, the corresponding donor patient featured a poor reduction in tumor mass following neo-adjuvant radiotherapy, while the tumor biopsies of patients who showed an optimal response to the radiotherapy produced CSC cultures with a low radiosensitivity threshold.

Hence, the in vitro data could help adapt the neoadjuvant radio treatment approach to improve clinical outcomes and reduce toxicity for patients with CRC.

Furthermore, in most CRC cases, in vitro radiotherapy response tests can be quickly performed, demonstrating the ability of this model to produce treatment suggestions in a clinically acceptable time frame. In conclusion, the assessment of the in vitro based model predicted CRC patient responses to radiotherapy treatment and could be developed as a powerful diagnostic tool for CRC treatment.

## 4. Materials and Methods

A complete study diagram is included as Supplementary Materials in Figure S1.

### 4.1. Patient Enrolment and Primary Human Tumor Collection

Newly diagnosed T3 or T4 colorectal cancer patients who were eligible for neo-adjuvant radiotherapy and subsequent surgical resection were enrolled at the Mediterranean Institute of Oncology (IOM, Viagrande, Italy). T2 patients eligible for neo adjuvant therapy, according to ESMO indication (mesorectal excision impaired by the state of the circumferential resection margin) were also enrolled.

Rectal cancer biopsy samples were obtained before the administration of neo-adjuvant radiotherapy. Eight weeks after neo-adjuvant radiotherapy, the individual responses to therapy were evaluated via CT-scan and Magnetic Resonance, and the patients were classified.

Table 2 reports the clinical characteristics of the involved patients. No evident correlation between the clinical characteristics and the ability to generate growing cultures was observed.

The study is compliant with all relevant ethical regulations involving human participants and was approved by the Institutional Review Board protocol (project ID code: 157_1 of 20 February 2017, IOM Institutional Review Board). Informed consent was obtained from all subjects.

### 4.2. CSC Isolation and Culture Establishment

Seven fresh human colorectal cancer biopsies were obtained in accordance with the standards of the ethics committee on human experimentation of the IOM. Biopsy samples were collected before the administration of neo-adjuvant radiotherapy. CSC isolation from the biopsies was performed as previously described [22,47]. Briefly, for dissociation, biopsies were first extensively washed in PBS (GIBCO, Thermo Fisher Scientific, Carlsbad, CA, USA) and then subjected to mechanical and enzymatic digestion with Collagenase type II (Thermo Fisher Scientific, Waltham, MA, USA) and DNAse I (Roche Diagnostics, Indianapolis, IN, USA) at 37 °C for 1 h. The cell suspension was then filtered through a 100 μm nylon sieve and the cell pellet was resuspended in a CSC medium for spheroid growth (Tumorsphere Medium XF, PromoCell, Heidelberg, Germany), plated in ultra-low attachment tissue culture flasks (Corning Costar, Cambridge, MA, USA), and incubated at 37 °C under a humidified atmosphere of 5% CO_2_. Every 2/3 days, half of the culture medium was refreshed. In these stringent culture conditions, immature cells grew slowly and formed non-adherent clusters, called tumor spheres, while non-malignant cells or differentiated cells died. Tumor spheres became evident after a variable length of time, ranging from 5 to 7 days to 3 weeks. Regular culture splitting (1:2) was usually needed after 3–6 weeks from isolation. Spheroids were weekly subjected to mechanical or enzymatic dissociation via incubation for 10 min at RT with the Accutase enzyme (GIBCO).

From a total of 7 surgical samples, 4 CSC lines were established and validated to determine their ability to generate tumors in mice, and 3 samples did not generate growing in vitro cultures (1 culture was affected by bacterial contamination, while 2 did not expand in the culture medium, remaining in a quiescent state for several months).

### 4.3. Evaluation of Stem Cell Marker Expression by Flow Cytometry

The expression of stem cell markers was evaluated by flow cytometry analysis using FACSAria II (Becton Dickinson (BD), Franklin Lakes, NJ, USA). Single cells dissociated from spheroids were incubated with the appropriate dilution of a specific antibody: anti CD133-PE (Miltenyi Biotec, Bergisch Gladbach, Germany) and anti CD44-FITC (BD Biosciences, San Jose, CA, USA). Unstained cells were used as the negative control.

Cell stemness was also evaluated by using an ADEFLUOR KIT (STEMCELL Technologies, Vancouver, BC, Canada) according to the manufacturer’s instructions. The ALDEFLUOR™ reagent system is a non-immunological method to identify stem/progenitor cells by their aldehyde dehydrogenase (ALDH) activity.

### 4.4. In Vitro Cell Irradiation

CSCs were seeded into 35 × 10 mm dishes (Corning, NY, USA) and reached approximately 80% confluency at the time of irradiation.

A system for the in vitro irradiation of CSCs and custom-designed irradiation geometry were developed. This system utilizes the same equipment used for patient treatments. To simulate the flow of radiation beams through human tissues before reaching the target tumor area, a single dish containing cultured CSCs was inserted into a custom-built phantom made of plexiglass, a material similar to water, which is the main component of human tissue. The dish was housed within a niche created inside the phantom to place the cells at the radiation isocenter.

The phantom containing the colorectal CSCs was irradiated through a Varian Novalis-TrueBeam STx linear accelerator, which is able to perform stereotactic treatments with very high precision. This radiotherapy equipment uses the high dose rate Flattening Filter Free (FFF) technique and a High Definition Multilamellar Collimator (MLC); with a minimum leaf size at the isocenter of 2.5 mm, this device is specifically designed to treat small lesions. A fractionated dose of 25 Gy (5 daily fractions of 5 Gy) was administered [43] in combination with different dose rates (600 MU/min, 1400 MU/min, and 2400 MU/min) to assess the eventual impact of dose rates on apoptosis and cell viability. The plan consisted of two opposed photon beams of 8 × 8 cm^2^ defined at the machine isocenter located at the center of the niche containing the plate.

After irradiation, cells were incubated at 37 °C in a humidified atmosphere of 5% CO_2_ for a further 24 h, 48 h, 72 h, 7 d, and 14 d, respectively, and then analyzed for cell viability, apoptosis, and the ability to give rise to new clones.

### 4.5. Evaluation of Cell Viability

The cell viability assay was performed using a CellTiter96^®^ Aqueous One Solution Cell Proliferation Assay Kit (Promega, Madison, WI, USA) according to the manufacturer’s instructions. The fluorescence signal was detected with Synergy HT (Biotek Instruments Inc., Winooski, VT, USA) at 24 h, 48 h, 72 h, 7 d, and 14 d.

### 4.6. Evaluation of Apoptosis

Annexin V staining of the phosphatidylserine (PS) in the outer surface of the cellular membrane is a widely used assay for studying cellular apoptosis, as an increase in PS staining is directly connected with early apoptosis. Here, 1 × 10^5^ cells for each sample were stained at 24 h, 48 h, 72 h, 7 d, and 14 d, with Annexin V fluorescein isothiocyanate (FITC) at a final concentration of 0.375 μg/mL (BD Biosciences, San Jose, CA, USA), according to the manufacturer’s instructions. To distinguish between early apoptotic cells with intact cellular membranes and necrotic or late-apoptotic cells, 1 μg of propidium iodide (PI) was added to each sample. Cytometric analysis was performed with a FACS-Aria II flow cytometer (BD Biosciences, San Jose, CA, USA). For each measurement, 1 × 10^4^ cells were counted, and the results were analyzed. Three replicates were analyzed for each CSC line in each condition assessed.

### 4.7. Single-Cell Cloning

CSCs were dissociated with Accutase (Gibco, Carlsbad, CA, USA) and then resuspended in a fresh medium to generate a single-cell suspension with a density of 10 cells/mL. Then, 200 μL of the single-cell suspension was dispensed into each well of a 96-well non-treated plate. The day after plating, only wells that contained 1 viable cell were selected, excluding wells with no cells or more than one cell. Single-cell cultures were maintained in a medium and were checked after 14 days to ascertain their clonogenic potential.

### 4.8. In Vivo Procedures

All animal procedures were performed according to the Italian national animal experimentation guidelines (D.L.116/92) upon approval from the experimental protocol by the Italian Ministry of Health’s Animal Experimentation Committee. This study used 4-to-6-week-old female NOD. Cg-Prkdc^scid^ Il2rg^tm1Wjl^/SzJ (NSG) mice (The Jackson Laboratory, Bar Harbor, ME, USA).

### 4.9. In Vivo Evaluation of CSCs’ Tumor Initiating Capabilities

The main feature of stem cells is their ability, once implanted in a recipient mouse, to reproduce a tumor with the same phenotype of the original one. To this end, 5 × 10^5^ cells were resuspended in 100 μL of a 1:1 growth medium/Matrigel (BD Biosciences, San Jose, CA, USA) solution, and the cell suspension was injected subcutaneously into the flank of the animal. For each CRC cell line, 5 replicates of xenotransplants were performed. For all 4 lines, a tumor mass was detectable within 3–5 weeks in at least 3 out of 5 mice. As soon as the tumor mass reached a diameter of 10 mm, xenografts were explanted, and one-half of the mass was formalin-fixed, paraffin-embedded, and processed for histology to evaluate the tumor phenotype in comparison with the parental human tumor. The other portion of tumors was dissociated into single cells that were seeded in a tumor sphere medium and expanded to be assayed once again for stemness markers (CD44 and CD133 expression, ALDH activity).

### 4.10. Histology and Immunohistochemistry

Tumors were fixed with 10% formalin and paraffin-embedded for histological analysis. Three-micrometer-thick sections were cut with a microtome and automatically stained with hematoxylin-eosin (Ventana Symphony Stainer, JMD Histology and Histologistics Inc., Dudley, MA, USA).

The presence of colon adenocarcinoma was also evaluated via immunohistochemical analysis. Three-micrometer-thick sections were cut from the FFPE blocks. Anti-human CK20 (clone SP33) and CDX2 (clone EPR2764Y) rabbit monoclonal primary antibodies (Ventana, Roche Diagnostic, Basel, Switzerland) were used for the analysis. Slides were incubated using the BenchMark ULTRA platform, and an OptiView DAB IHC Detection Kit (Roche Diagnostic, Basel, Switzerland) was used to detect protein expression. Tissues were counterstained with Hematoxylin II (Roche Diagnostic, Basel, Switzerland) for 4 min. The control of immunostaining specificity was performed by omitting the primary antibody.

### 4.11. In Vivo Tumor Irradiation and Evaluation of Therapeutic Response

4- to 6-week-old female NSG mice (The Jackson Laboratory, Bar Harbor, ME, USA) were randomly assigned into 4 groups, one group for each CSC line. Each group was formed from 11 mice. For each line, the CRC-SCs were resuspended in 100 μL of a 1:1 growth medium/Matrigel, and 5 × 10^5^ cells were injected subcutaneously into the flank of the animal [48]. Tumor growth was measured twice weekly via an external digital caliper, and volumes were calculated using the following formula: π/6 × d^2^ × D, where d and D represent shorter and longer tumor measurements, respectively. When tumors reached a dimension of 100–150 mm^3^, the mice were randomly assigned to the control (3 mice/group) and treatment groups (8 mice/group). On the day of the radio treatment, mice belonging to the treatment group were moved, one by one, from the cage into a plexiglass box, where an anesthetic gas containing Vetflurane (Virbac, Barcelona, Spain) was insufflated. All the following procedures, including image acquisition, contouring, elaboration of the treatment plan, and the same radio-treatment, were performed with the mice inserted and immobilized inside the plexiglass cage. Here, mice were first subjected to a computed tomography (CT) scan, and the CT images were the sent to the treatment planning system (TPS) dedicated to stereotactic radiotherapy treatments. Here, contouring of the volumes of interest was performed, including the target volume, spinal cord, heart, lungs, and bowels. At this point, the treatment plan was elaborated. This plan consisted of two non-coplanar dynamic conformal arcs with the optimized opening of MLC leaves based on dose constraints established during planning. Plan evaluation was performed carefully while observing the dose distributions on each CT image and the dose–volume histograms to assess the radiation dose that reached the target and the neighboring organs. To set-up the verification, we used the image-guided radiotherapy system “ExacTrac X-Ray 6D”, through using which it was possible to carry out pre-positioning through the infrared system and positioning using the X-Ray imaging system. Irradiation of the mice was performed by delivering 5 Gy for 5 consecutive days, for a total dose of 25 Gy at a maximum dose rate of 2400 MU/min using the 10 MV FFF photon beam produced by the Varian Novalis-TrueBeam STx linear accelerator.

Control animals were inoculated but not treated. Thirty days after treatment, a CT scan was performed to verify the tumor dimensions.

This study was performed in accordance with the ethical statement established by Italian law (Decreto legislativo 4 marzo 2014, n. 26). This study was authorized by the Italian Ministry of Health with the code 0D183.2.

### 4.12. Patient Study

The patients enrolled in this observational study, whose isolated cells were used for in vitro and in vivo studies, were treated according to the actual therapeutic protocol. At the end of the treatment, the individual responses to therapy were evaluated via CT scan, Magnetic Resonance, and colonoscopy, and the data were compared with the in vitro results.

The effectiveness of the treatment was also assessed using the tumor regression grade on surgical specimen by histological analysis. Tumor regression grading (TRG) is used to categorize the number of regressive changes after the treatment, estimating the percentage of residual tumor in relation to the previous tumor site.

### 4.13. Statistical Analysis

Quantitative endpoints (MTS, Annexin V and subcutaneous tumor masses volume) measured at different timepoints were evaluated between groups of the treatments and controls using a repeated measures ANOVA (RMAN). Statistical analysis was performed using the R statistical environment (R Core Team (2019). R is a language and environment for statistical computing. R Foundation for Statistical Computing, Vienna, Austria. URL https://www.R-project.org/).

## 5. Conclusions

In this study, we demonstrated the intrinsic individual sensitivity of CSC to radiotherapy. This sensitivity profile was conserved under both in vitro and in vivo treatments in all the assessed samples, thus indicating that the subcutaneous tumors inherited this feature from their parental cells. Sensitive CSC were observed to be obtained by patients showing an optimal response to the therapeutic protocol. On the other hand, cells isolated from the biopsies of radioresistant tumors give rise to radioresistant CSC cultures. This suggest that the effect exerted by the administration of radiotherapy in vitro may be useful to predict the outcome of the treatment in donor patients. CSC cultures may be rapidly established by most of the samples obtained by LARC patients. These cultures can be rapidly expanded and employed for their sensitivity by using the described in vitro treatment protocol. The possibility to employ primary sphere-forming cultures without the need to use mice models facilitates the translation of the model, thereby reducing both the time and costs needed. We also observed that some samples did not yield CSC cultures. The reason for this requires additional investigations. The limited size of the patient cohort also highlights the preliminary nature of the presented study. Nevertheless, the novelty of the approach may help deepen the suitability of the model for translation into a clinical setting through a broader study. The proposed approach may be suitable thanks to its time frame, which may fit the current therapeutic settings for LARC. In conclusion, assessment of the in vitro based model predicted CRC patient responses to radiotherapy treatment, so this model could be developed as a powerful diagnostic tool for CRC treatment.

## Figures and Tables

**Table 1:** Typical stem-cell markers evaluated on tumor sphere cultures.

	CSC Line 1	CSC Line 2	CSC Line 3	CSC Line 4
CD44	21%	70.70%	76%	21.40%
CD133	21.50%	25.50%	25.70%	21.75%
aldehyde dehydrogenase (ALDH)	MEDIUM	MEDIUM	MEDIUM	MEDIUM

**Table 2:** Clinical characteristics of the patients enrolled. Patients for whom cell lines were not obtained are reported with gray background.

CRC Line	Age	Sex	Diagnosis	T	N	M	Stage	Treatment	TRG	Mutation Status		
										KRAS	NRAS	BRAF
1	60	M	adenocarcinoma	3	2	X	3B	5FU + 50GY RADIO NEOADJ	2	WT	WT	WT
2	50	M	adenocarcinoma	3	0	0	2A	5FU + 50GY RADIO NEOADJ	1	WT	WT	WT
3	46	F	adenocarcinoma	3	1	0	3B	5FU + 50GY RADIO NEOADJ	1	MUT	WT	WT
4	78	F	adenocarcinoma	2	0	0	2	5FU + 50GY RADIO NEOADJ	2	WT	WT	WT
NE1	68	F	adenocarcinoma	3	1	0	3B	5FU + 50GY RADIO NEOADJ	0	WT	WT	WT
NE2	67	F	adenocarcinoma	3	1	0	3B	5FU + 50GY RADIO NEOADJ	0	WT	WT	WT
NE3	61	M	adenocarcinoma	3	1	0	3B	5FU + 50GY RADIO NEOADJ	3	WT	MUT	WT

## Supplementary Materials

The following are available online at https://www.mdpi.com/2072-6694/12/12/3672/s1, Figure S1: Study diagram.

## References

[1] Bray F., Ferlay J., Soerjomataram I., Siegel R.L., Torre L.A., Jemal A. (2018). Global cancer statistics 2018: GLOBOCAN estimates of incidence and mortality worldwide for 36 cancers in 185 countries. CA Cancer J. Clin..

[2] Fedirko V., Tramacere I., Bagnardi V., Rota M., Scotti L., Islami F., Negri E., Straif K., Romieu I., La Vecchia C. (2011). Alcohol drinking and colorectal cancer risk: An overall and dose-response meta-analysis of published studies. Ann. Oncol..

[3] Liang P.S., Chen T.-Y., Giovannucci E. (2009). Cigarette smoking and colorectal cancer incidence and mortality: Systematic review and meta-analysis. Int. J. Cancer.

[4] Renehan A.G., Tyson M., Egger M., Heller R.F., Zwahlen M. (2008). Body-mass index and incidence of cancer: A systematic review and meta-analysis of prospective observational studies. Lancet.

[5] Samad A.K.A., Taylor R.S., Marshall T., Chapman M.A.S. (2005). A meta-analysis of the association of physical activity with reduced risk of colorectal cancer. Color. Dis..

[6] Modan B., Barell V., Lubin F., Modan M., Greenberg R.A., Graham S. (1975). Low-Fiber Intake as an Etiologic Factor in Cancer of the Colon 2. J. Natl. Cancer Inst..

[7] Chan D.S.M., Lau R., Aune D., Vieira R., Greenwood D.C., Kampman E., Norat T. (2011). Red and Processed Meat and Colorectal Cancer Incidence: Meta-Analysis of Prospective Studies. PLoS ONE.

[8] Heald R., Moran B. (1998). Embryology and anatomy of the rectum. Semin. Surg. Oncol..

[9] Iacopetta B. (2002). Are there two sides to colorectal cancer?. Int. J. Cancer.

[10] Kapiteijn E., Marijnen C.A., Nagtegaal I.D., Putter H., Steup W.H., Wiggers T., Rutten H.J., Pahlman L., Glimelius B., Van Krieken J.H.J. (2001). Preoperative Radiotherapy Combined with Total Mesorectal Excision for Resectable Rectal Cancer. N. Engl. J. Med..

[11] Sauer R., Becker H., Hohenberger W., Rödel C., Wittekind C., Fietkau R., Martus P., Tschmelitsch J., Hager E., Hess C.F. (2004). Preoperative versus Postoperative Chemoradiotherapy for Rectal Cancer. N. Engl. J. Med..

[12] Bosset J.-F., Collette L., Calais G., Mineur L., Maingon P., Radosevic-Jelic L., Daban A., Bardet E., Beny A., Ollier J.-C. (2006). Chemotherapy with Preoperative Radiotherapy in Rectal Cancer. N. Engl. J. Med..

[13] Couwenberg A.M., Burbach J.P.M., Van Grevenstein W.M., Smits A.B., Consten E.C., Schiphorst A.H.W., Wijffels N.A., Heikens J.T., Intven M.P.W., Verkooijen H.M. (2018). Effect of Neoadjuvant Therapy and Rectal Surgery on Health-related Quality of Life in Patients With Rectal Cancer During the First 2 Years After Diagnosis. Clin. Color. Cancer.

[14] Glynne-Jones R., Debus J. (2001). Improving Chemoradiotherapy in Rectal Cancer. Oncologist.

[15] Bonnet D., Dick J.E. (1997). Human acute myeloid leukemia is organized as a hierarchy that originates from a primitive hematopoietic cell. Nat. Med..

[16] Al-Hajj M., Wicha M.S., Benito-Hernandez A., Morrison S.J., Clarke M.F. (2003). Prospective identification of tumorigenic breast cancer cells. Proc. Natl. Acad. Sci. USA.

[17] Singh S.K., Hawkins C., Clarke I.D., Squire J.A., Bayani J., Hide T., Henkelman R.M., Cusimano M.D., Dirks P.B. (2004). Identification of human brain tumour initiating cells. Nat. Cell Biol..

[18] Patrawala L., Calhoun T., Schneiderbroussard R., Li H., Bhatia B., Tang S., Reilly J., Chandra D., Zhou J., Claypool K. (2006). Highly purified CD44+ prostate cancer cells from xenograft human tumors are enriched in tumorigenic and metastatic progenitor cells. Oncogene.

[19] Matsui W., Huff C.A., Wang Q., Malehorn M.T., Barber J., Tanhehco Y., Smith B.D., Civin C.I., Jones R.J. (2004). Characterization of clonogenic multiple myeloma cells. Blood.

[20] Dalerba P., Clarke M.F. (2007). Cancer stem cells and tumor metastasis: First steps into uncharted territory. Cell Stem Cell.

[21] Schatton T., Murphy G.F., Frank N.Y., Yamaura K., Waaga-Gasser A.M., Gasser M., Zhan Q., Jordan S., Duncan L.M., Weishaupt C. (2008). Identification of cells initiating human melanomas. Nat. Cell Biol..

[22] Ricci-Vitiani L., Lombardi D.G., Pilozzi E., Biffoni M., Todaro M., Peschle C., De Maria R. (2007). Identification and expansion of human colon-cancer-initiating cells. Nat. Cell Biol..

[23] Eramo A., Lotti F., Sette G., Pilozzi E., Biffoni M., Di Virgilio A., Conticello C., Ruco L., Peschle C., De Maria R. (2008). Identification and expansion of the tumorigenic lung cancer stem cell population. Cell Death Differ..

[24] Todaro M., Iovino F., Eterno V., Cammareri P., Gambara G., Espina V., Gulotta G., Dieli F., Giordano S., De Maria R. (2010). Tumorigenic and Metastatic Activity of Human Thyroid Cancer Stem Cells. Cancer Res..

[25] Giuffrida R., Adamo L., Iannolo G., Vicari L., Giuffrida D., Eramo A., Gulisano M., Memeo L., Conticello C. (2016). Resistance of papillary thyroid cancer stem cells to chemotherapy. Oncol. Lett..

[26] Ward R.J., Dirks P.B. (2007). Cancer Stem Cells: At the Headwaters of Tumor Development. Annu. Rev. Pathol. Mech. Dis..

[27] Garza-Treviño E.N., Said-Fernández S., Martínez-Rodríguez H.G. (2015). Understanding the colon cancer stem cells and perspectives on treatment. Cancer Cell Int..

[28] Asfaha S., Hayakawa Y., Muley A., Stokes S., Graham T.A., Ericksen R.E., Westphalen C.B., Von Burstin J., Mastracci T.L., Worthley D.L. (2015). Krt19+/Lgr5− Cells Are Radioresistant Cancer-Initiating Stem Cells in the Colon and Intestine. Cell Stem Cell.

[29] Kemper K., Prasetyanti P.R., De Lau W., Rodermond H., Clevers H., Medema J.P. (2012). Monoclonal Antibodies Against Lgr5 Identify Human Colorectal Cancer Stem Cells. Stem Cells.

[30] Hirsch D., Barker N., McNeil N., Hu Y., Camps J., McKinnon K., Clevers H., Ried T., Gaiser T. (2014). LGR5 positivity defines stem-like cells in colorectal cancer. Carcinogenesis.

[31] Wilson B.J., Schatton T., Frank M., Frank N.Y. (2011). Colorectal Cancer Stem Cells: Biology and Therapeutic Implications. Curr. Color. Cancer Rep..

[32] Yan Y., Zuo X., Wei D. (2015). Concise Review: Emerging Role of CD44 in Cancer Stem Cells: A Promising Biomarker and Therapeutic Target. Stem Cells Transl. Med..

[33] Todaro M., Gaggianesi M., Catalano V., Benfante A., Iovino F., Biffoni M., Apuzzo T., Sperduti I., Volpe S., Cocorullo G. (2014). CD44v6 Is a Marker of Constitutive and Reprogrammed Cancer Stem Cells Driving Colon Cancer Metastasis. Cell Stem Cell.

[34] Medema J.P. (2013). Cancer stem cells: The challenges ahead. Nat. Cell Biol..

[35] Vermeulen L., Todaro M., Melo F.D.S.E., Sprick M.R., Kemper K., Alea M.P., Richel D.J., Stassi G., Medema J.P. (2008). Single-cell cloning of colon cancer stem cells reveals a multi-lineage differentiation capacity. Proc. Natl. Acad. Sci. USA.

[36] Hanahan D., Weinberg R.A. (2000). The Hallmarks of Cancer. Cell.

[37] Kozovska Z., Gabrisova V., Kucerova L. (2014). Colon cancer: Cancer stem cells markers, drug resistance and treatment. Biomed. Pharmacother..

[38] Pearce D.J., Taussig D., Simpson C., Allen K., Rohatiner A.Z., Lister T.A., Bonnet D. (2005). Characterization of Cells with a High Aldehyde Dehydrogenase Activity from Cord Blood and Acute Myeloid Leukemia Samples. Stem Cells.

[39] Jelski W., Chrostek L., Szmitkowski M. (2007). The Activity of Class I, II, III, and IV of Alcohol Dehydrogenase Isoenzymes and Aldehyde Dehydrogenase in Pancreatic Cancer. Pancreas.

[40] Ginestier C., Hur M.H., Charafe-Jauffret E., Monville F., Dutcher J., Brown M., Jacquemier J., Viens P., Kleer C.G., Liu S. (2007). ALDH1 Is a Marker of Normal and Malignant Human Mammary Stem Cells and a Predictor of Poor Clinical Outcome. Cell Stem Cell.

[41] Ucar D., Cogle C.R., Zucali J.R., Ostmark B., Scott E.W., Zori R., Gray B.A., Moreb J.S. (2009). Aldehyde dehydrogenase activity as a functional marker for lung cancer. Chem. Interact..

[42] Kozovska Z., Patsalias A., Bajzik V., Durinikova E., Demkova L., Jargasova S., Smolkova B., Plava J., Kucerova L., Matuskova M. (2018). ALDH1A inhibition sensitizes colon cancer cells to chemotherapy. BMC Cancer.

[43] Folkesson J., Birgisson H., Pahlman L., Cedermark B., Glimelius B., Gunnarsson U. (2005). Swedish Rectal Cancer Trial: Long Lasting Benefits From Radiotherapy on Survival and Local Recurrence Rate. J. Clin. Oncol..

[44] Glynne-Jones R., Wyrwicz L., Tiret E., Brown G., Rödel C., Cervantes A., Arnold D. (2017). Rectal cancer: ESMO Clinical Practice Guidelines for diagnosis, treatment and follow-up. Ann. Oncol..

[45] Van Der Valk M.J.M., Hilling D.E., Bastiaannet E., Kranenbarg E.M.-K., Beets G.L., Figueiredo N.L., Habr-Gama A., Perez R.O., Renehan A.G., Van De Velde C.J.H. (2018). Long-term outcomes of clinical complete responders after neoadjuvant treatment for rectal cancer in the International Watch & Wait Database (IWWD): An international multicentre registry study. Lancet.

[46] Renehan A.G., Malcomson L., Emsley R., Gollins S., Maw A., Myint A.S., Rooney P.S., Susnerwala S., Blower A., Saunders M. (2016). Watch-and-wait approach versus surgical resection after chemoradiotherapy for patients with rectal cancer (the OnCoRe project): A propensity-score matched cohort analysis. Lancet Oncol..

[47] Fiori M.E., Villanova L., De Maria R. (2017). Cancer stem cells: At the forefront of personalized medicine and immunotherapy. Curr. Opin. Pharmacol..

[48] De Angelis M.L., Zeuner A., Policicchio E., Russo G., Bruselles A., Signore M., Vitale S., De Luca G., Pilozzi E., Boe A. (2016). Cancer Stem Cell-Based Models of Colorectal Cancer Reveal Molecular Determinants of Therapy Resistance. Stem Cells Transl. Med..

