# Supplementary figures and images for: Radiosensitivity of Cancer Stem Cells Has Potential Predictive Value for Individual Responses to Radiotherapy in Locally Advanced Rectal Cancer
Source: Cancers (Basel). 2020 Dec 7;12(12):3672. doi: 10.3390/cancers12123672 (PMC7762426; doi:10.3390/cancers12123672)

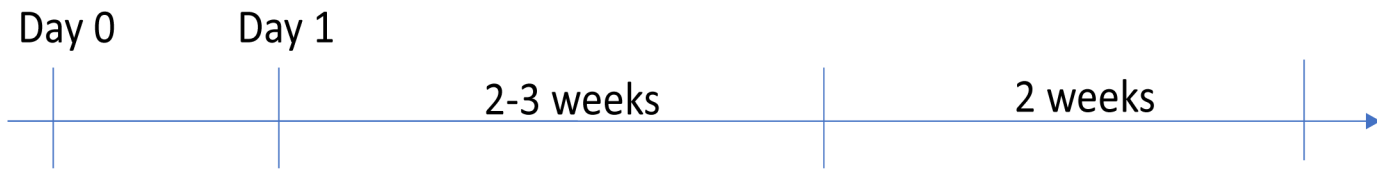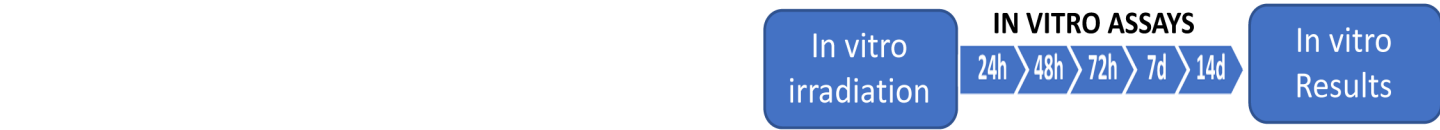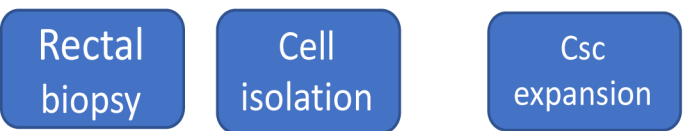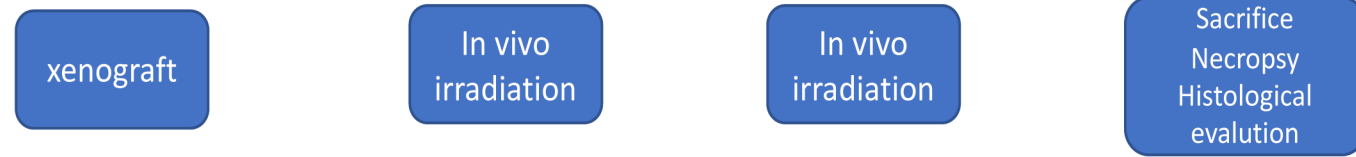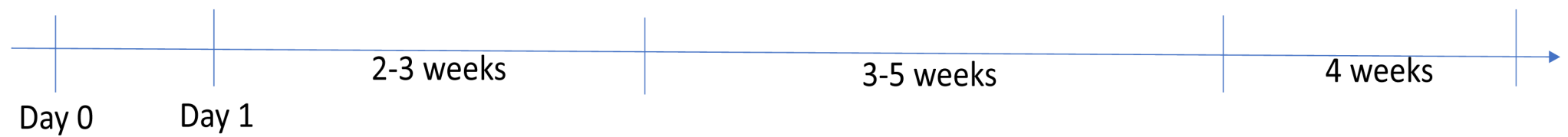

Supplement: Supplementary file 1 [file cancers-12-03672-s001.pdf]
